# Acromegaly Disease Control Maintained After Switching From Injected Somatostatin Receptor Ligands to Oral Paltusotine

**DOI:** 10.1210/clinem/dgae385

**Published:** 2024-06-03

**Authors:** Mônica R Gadelha, Alessandra Casagrande, Christian J Strasburger, Martin Bidlingmaier, Peter J Snyder, Mirtha A Guitelman, Cesar L Boguszewski, Michael Buchfelder, Ilan Shimon, Gerald Raverot, Miklós Tóth, Emese Mezősi, Mirjana Doknic, Xiaolin Fan, David Clemmons, Peter J Trainer, R Scott Struthers, Alan Krasner, Beverly M K Biller

**Affiliations:** Neuroendocrinology Research Center/Endocrinology Division, Medical School and Hospital Universitario Clementino Fraga Filho, Universidade Federal do Rio de Janeiro, Rio de Janeiro CEP 21941-913, Brazil; Crinetics Pharmaceuticals, Inc., San Diego, CA 92121, USA; Department of Medicine for Endocrinology and Metabolic Disorders, Charité Universitaetsmedizin, Berlin 10117, Germany; Neuroendocrine Research Group, Department of Internal Medicine, Ludwig-Maximilians University, Munich 80336, Germany; Division of Endocrinology, Diabetes and Metabolism, Department of Medicine, University of Pennsylvania, Philadelphia, PA 19104, USA; División Endocrinología, Hospital Carlos G. Durand, Buenos Aires 1405, Argentina; Department of Internal Medicine, Endocrine Division (SEMPR), University Hospital, Federal University of Paraná, Curitiba 80030-110, Brazil; Neurosurgery Department, University Hospital Erlangen, Erlangen 91054, Germany; Rabin-Beilinson Medical Center, Institute of Endocrinology, Petach Tikva 4941492, Israel; Faculty of Medical and Health Sciences, School of Medicine, Tel Aviv University, Tel Aviv 6997801, Israel; Endocrinology Department, Reference Center for Rare Pituitary Diseases HYPO, “Groupement Hospitalier Est” Hospices Civils de Lyon, Lyon Cedex 69002, France; Department of Internal Medicine and Oncology, Semmelweis University, Budapest 1083, Hungary; Department of Medicine, University of Pécs Medical School, Pécs 7624, Hungary; Neuroendocrine Department, Clinic for Endocrinology, Diabetes and Metabolic Diseases, University Clinical Center of Serbia, Faculty of Medicine, University of Belgrade, Belgrade 11000, Serbia; Crinetics Pharmaceuticals, Inc., San Diego, CA 92121, USA; Department of Medicine, University of North Carolina School of Medicine, Chapel Hill, NC 27514, USA; Crinetics Pharmaceuticals, Inc., San Diego, CA 92121, USA; Crinetics Pharmaceuticals, Inc., San Diego, CA 92121, USA; Crinetics Pharmaceuticals, Inc., San Diego, CA 92121, USA; Neuroendocrine and Pituitary Tumor Clinical Center, Massachusetts General Hospital, Boston, MA 02114, USA

**Keywords:** acromegaly, paltusotine, IGF-I, somatostatin, somatostatin receptor 2, clinical trial

## Abstract

**Context:**

Paltusotine is a nonpeptide selective somatostatin receptor 2 agonist in development as once-daily oral treatment for acromegaly.

**Objective:**

To evaluate the efficacy and safety of paltusotine in the treatment of patients with acromegaly previously controlled with injected somatostatin receptor ligands (SRLs).

**Methods:**

This phase 3, randomized, double-blind, placebo-controlled trial enrolled adults with acromegaly who had IGF-I ≤1.0 times the upper limit of normal (×ULN) while receiving a stable dose of depot octreotide or lanreotide. Patients were switched from injected SRLs and randomized to receive paltusotine or placebo orally for 36 weeks. The primary endpoint was proportion of patients maintaining IGF-I ≤1.0× ULN. Secondary endpoints were change in IGF-I level, change in Acromegaly Symptom Diary score, and maintenance of mean 5-sample GH <1.0 ng/mL.

**Results:**

The primary endpoint was met: 83.3% (25/30) of patients receiving paltusotine and 3.6% (1/28) receiving placebo maintained IGF-I ≤1.0× ULN (odds ratio, 126.53; 95% CI, 13.73->999.99; *P* < .0001). Paltusotine was also superior to placebo for all secondary endpoints: mean (± SE) change in IGF-I of 0.04 ± 0.09× ULN vs 0.83 ± 0.1× ULN (*P* < .0001); mean (± SE) change in Acromegaly Symptom Diary score of −0.6 ± 1.5 vs 4.6 ± 1.6 (*P* = .02); mean GH maintained at <1.0 ng/mL in 20/23 (87.0%) vs 5/18 (27.8%) patients (odds ratio, 16.61; 95% CI, 2.86-181.36; *P* = .0003). The most common adverse events were acromegaly symptoms and gastrointestinal effects characteristic of SRLs.

**Conclusion:**

Replacement of injected SRLs by once-daily oral paltusotine was effective in maintaining both biochemical and symptom control in patients with acromegaly and was well tolerated.

Acromegaly is a rare chronic disease almost always caused by a pituitary adenoma that secretes excess GH, which results in overproduction of IGF-I (1, 2). Although surgical resection of the GH-producing pituitary tumor is the preferred initial therapy (3-5), approximately 45% of patients undergoing this treatment do not achieve GH and IGF-I control with surgery alone (6).

The somatostatin receptor ligands (SRLs) octreotide and lanreotide are peptide analogs that act predominately via somatostatin receptor 2 (SST2), with additional weak-to-moderate affinity for SST3 and SST5, to inhibit GH secretion, and, consequently, reduce IGF-I levels (7, 8). SRLs are approved for the treatment of acromegaly, gastroenteropancreatic neuroendocrine tumors, and carcinoid syndrome (9). Monthly injected SRLs are currently first-line medical treatment for acromegaly (10) but are associated with substantial burdens that include injection site pain and reactions (11, 12), suboptimal accuracy of drug delivery (13, 14), variable biochemical and symptom control across the dosing interval (12, 15), and, often, administration by health care professionals, necessitating repeat office visits.

Paltusotine is a novel, highly selective, nonpeptide SST2 agonist being developed as an oral treatment for patients with acromegaly or carcinoid syndrome (16, 17). Using iterative medicinal chemistry and subsequent structure–activity relationship studies, paltusotine was selected from a series of newly synthesized compounds based on specific target characteristics (17). In addition to SST2 receptor selectivity (which is >4000-fold relative to other SST receptor subtypes), paltusotine was shown to be associated with efficient absorption through the gastrointestinal tract, allowing for oral administration, a pharmacokinetic profile consistent with once-daily dosing, and a low risk of drug–drug interactions (16, 17).

An open-label, single-arm, phase 2 study explored the effects of paltusotine in patients with acromegaly who switched from SRL depot injections and showed that IGF-I and GH levels were maintained and patient-reported symptom burden remained stable (18). The current randomized, double-blind, placebo-controlled, phase 3 Paltusotine Acromegaly THerapy Featuring a Non-invasive Daily Regimen (PATHFNDR-1; NCT04837040) trial evaluated the efficacy and safety of once-daily oral paltusotine in patients whose acromegaly was controlled with injected SRLs.

## Materials and Methods

### Study Design

**Figure 1.**
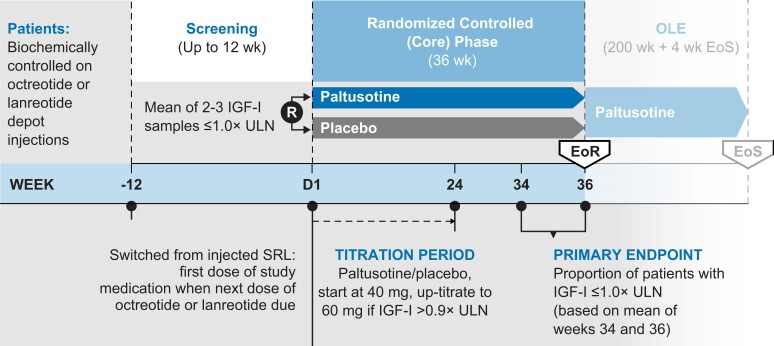
Study design.

Abbreviations: EoR, end of the randomized controlled phase; EoS, end of study; OLE, open-label extension; R, randomization; SRL, somatostatin receptor ligand; ULN, upper limit of normal.

PATHFNDR-1 was a multinational, phase 3, randomized, double-blind, placebo-controlled trial conducted at 30 centers in 13 countries. The study included a screening period, a core phase of up to 36 weeks of randomized treatment, and an open-label extension phase (currently ongoing; Fig. 1).

The study was conducted in accordance with the Guideline for Good Clinical Practice of the International Conference on Harmonisation, the ethical principles of the Declaration of Helsinki, and local regulatory guidelines. An institutional review board or independent ethics committee at each site approved the protocol, and all patients provided written informed consent.

### Patients

Patients were at least 18 years of age, had a confirmed diagnosis of acromegaly, and had been receiving a stable, effective dose of octreotide or lanreotide depot injections for at least 12 weeks. At screening, patients were required to have an IGF-I level less than or equal to 1.0 times the upper limit of normal (×ULN), based on an average of 2 or 3 separate measurements. Key exclusion criteria included pituitary surgery within 24 weeks before screening; any prior pituitary radiation; recent use of pasireotide (within 24 weeks) or pegvisomant, dopamine agonists, or short-acting SRLs (within 12 weeks); and poorly controlled diabetes (hemoglobin A1c ≥ 8.5%), cardiovascular, renal, or hepatic disease. Patients were excluded if the dose of injected octreotide was greater than 40 mg every 4 weeks, the dose of injected lanreotide was greater than 120 mg every 4 weeks, or the SRL dosing interval was less than 4 weeks. Oral estrogen was permitted if use began at least 12 weeks before screening.

### Randomization

Eligible patients were randomized (1:1) to paltusotine or placebo using an interactive voice/web response system and stratified by IGF-I level (<0.86× ULN or ≥0.86× ULN) and prior treatment (octreotide or lanreotide) via a fixed block randomization scheme. All investigators, trial personnel, and patients were unaware of the treatment assignments.

### Interventions

The first dose of study medication (paltusotine or matching placebo tablets) was administered when the next injection of octreotide or lanreotide would have been due. Patients were instructed to take study medication with water once daily in the morning, after an overnight fast of at least 6 hours, and then to wait an hour before eating (19). The starting dose of paltusotine was 40 mg/day, which was up-titrated to 60 mg/day during the first 24 weeks of the core phase if the most recent IGF-I level was > 0.9× ULN and tolerability was acceptable. Down-titration to as low as 20 mg/day was allowed at any time based on tolerability.

Per protocol, rescue medication (patient's prior injectable SRL) was to be administered if both of these criteria were met: 2 consecutive IGF-I levels ≥1.3× ULN at the highest dose of study medication (60 mg/day) and exacerbation of acromegaly clinical signs and symptoms as assessed by the investigator.

### Study Endpoints and Assessments

The primary endpoint was the proportion of patients maintaining IGF-I ≤1.0× ULN at the end of the core phase (mean of weeks 34 and 36). Secondary efficacy endpoints were change from baseline in IGF-I (measured as ×ULN) at the end of randomized treatment (week 36 if no rescue medication, or the last assessment before rescue); change from baseline in total Acromegaly Symptom Diary (ASD) score at the end of randomized treatment; and the proportion of patients with mean 5-sample GH <1.0 ng/mL at week 34 among those with mean 5-sample GH <1.0 ng/mL during screening. Exploratory endpoints included change in mean GH level at week 34; time to loss of biochemical control, defined as the time from randomization to first IGF-I >1.0× ULN for 2 consecutive visits (with study medication dose of 60 mg/day for ≥2 weeks); and the proportion of patients who received rescue therapy.

Serum IGF-I and GH were measured centrally using iSYS immunoassays (Immunodiagnostic Systems). Baseline IGF-I was calculated as the mean of screening and day 1 values. Because of the pulsatile nature of GH secretion, the mean value was determined from 5 fasting samples collected at least 30 minutes apart within a 3-hour period. Age- and sex-related reference standards for IGF-I were previously established using the iSYS assay and samples from more than 15 000 patients (20). The IGF-I assay used the National Institute for Biological Standards and Control recombinant standard code 02/254 and yielded intra-assay variability of 1.3% to 3.7%, inter-assay variability of 3.4% to 8.7%, and sensitivity of 8.8 ng/mL (20). The GH assay used National Institute for Biological Standards and Control recombinant standard code 98/574 and yielded intra-assay variability of 1.1% to 3.4%, inter-assay variability of 0.9% to 3.8%, and sensitivity of 0.04 ng/mL (21).

Acromegaly symptoms were evaluated using the ASD, a newly developed scale that includes 7 core symptoms (headache, joint pain, sweating, fatigue, leg weakness, swelling, numbness/tingling) plus additional items for sleep difficulty and short-term memory difficulty (22). Patients rated symptoms for the previous 24 hours from 0 (no symptom) to 10 (worst symptom). Each patient identified their most bothersome symptom at baseline. ASD total score was the sum of scores for the 7 core symptoms (range, 0-70).

Safety assessments included adverse effect (AE) monitoring, clinical laboratory tests, vital signs, electrocardiogram (every 4-8 weeks), and abdominal (gallbladder) ultrasonography (baseline and end of treatment). For each reported AE, the investigator was asked to assess whether it was considered a symptom of the patient's acromegaly or related to study medication. Treatment adherence was assessed at each study visit by tablet counts of returned study medication.

Pituitary tumor volume was calculated at baseline and end of treatment from magnetic resonance imaging scans performed locally in accordance with specified image acquisition standards and read centrally by an experienced neurosurgeon who was blinded to treatment assignment. Image analysis followed a stepwise procedure that began with determination of whether there was a visible adenoma and, if so, included measurement of the maximum diameters (height, width, depth) on coronal, sagittal, and, if needed, axial images. The image datasets were imported into iPlan software (BrainLab; Munich, Germany). On the planning station, the adenoma was manually delineated, and volumetric analysis was performed automatically. A virtual 3-dimensional adenoma was generated on the basis the of the “segmented” structure and used for follow-up comparison.

### Statistical Analysis

The planned sample size (26 patients per treatment group) was selected based on a 2-sample Fisher exact test with 2-sided alpha = .05, to provide at least 93% power to detect superiority of paltusotine over placebo, assuming rates of IGF-I ≤1.0× ULN of 70% and 20% for paltusotine and placebo, respectively.

The proportion of patients who met the primary endpoint was evaluated using an exact logistic regression model with categorical covariates of prior treatment (octreotide, lanreotide) and screening IGF-I level (<0.86× ULN, ≥0.86× ULN). Patients were considered to have met the primary endpoint if IGF-I was ≤1.0× ULN based on the average of weeks 34 and 36. If only 1 measurement was available (week 34 or 36), it was used to evaluate the primary endpoint. If study treatment was discontinued for any reason (including meeting protocol criteria for rescue therapy), patients were considered to have failed to meet the primary endpoint.

To control the family-wise type I error rate, a hierarchical testing procedure was used. If the primary analysis was significant (predefined alpha level, .05), secondary endpoints were analyzed in this order: change from baseline in IGF-I, change from baseline in ASD score, and proportion of patients with 5-sample mean GH <1.0 ng/mL at screening and week 34. Changes in IGF-I level and ASD score were evaluated using analysis of covariance. The proportion of patients who maintained 5-sample GH <1.0 ng/mL was analyzed using the same methodology as for the primary endpoint. Time to loss of biochemical control was determined using the Kaplan-Meier method. Sensitivity analyses were performed to evaluate the primary endpoint in patients who completed at least 34 weeks of treatment and in the per-protocol population (defined as randomized patients who received at least 1 dose of study medication, had no protocol deviations that would affect efficacy, and had at least 75% treatment compliance based on tablet counts).

## Results

### Patients

**Figure 2.**
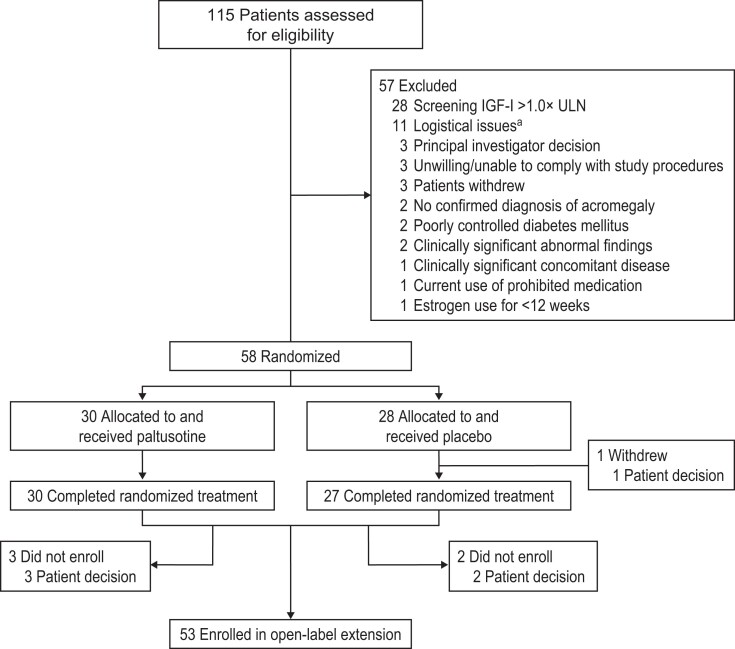
Patient disposition. ^a^Sponsor decision to discontinue participation of Russian study sites because of logistical study operation difficulties related to international conflict. Abbreviations: CYP, cytochrome P450; ULN, upper limit of normal.

The study was conducted from May 2021 through July 2023 (last core phase visit). Fifty-eight patients were randomized and received at least 1 dose of study medication (paltusotine n = 30; placebo n = 28). Fifty-seven (98.3%) patients completed the core phase (Fig. 2). Treatment groups were balanced for demographics and disease characteristics, except for a greater percentage of females in the placebo group and longer duration of acromegaly in the paltusotine group (Table 1). Pretrial use of high-dose octreotide, but not lanreotide, was more common in the paltusotine group. Baseline IGF-I and GH concentrations were comparable between treatment groups. In the paltusotine group, the final dose at the end of randomized treatment was 40 mg/day in 14 (46.7%) patients and 60 mg/day in 16 (53.3%) patients.

**Table 1.** Demographics and baseline characteristics by randomized treatment group

	Paltusotine (n = 30)	Placebo (n = 28)
Age, y, mean (SD)	55.9 (14.6)	53.9 (12.9)
Sex, n (%)		
Female	15 (50.0)	17 (60.7)
Male	15 (50.0)	11 (39.3)
Race, n (%)		
Asian	0	2 (7.1)
Black or African American	2 (6.7)	1 (3.6)
White	23 (76.7)	19 (67.9)
Multiple/Other	3 (10.0)	4 (14.3)
Unknown	2 (6.7)	2 (7.1)
Hispanic/Latino ethnicity, n (%)		
Yes	11 (36.7)	8 (28.6)
No	17 (56.7)	18 (64.3)
Unknown	2 (6.7)	2 (7.1)
Geographic region, n (%)		
Europe and Israel	13 (43.3)	12 (42.9)
South America	11 (36.7)	11 (39.3)
United States	6 (20.0)	5 (17.9)
Duration of acromegaly (since diagnosis), n (%)		
<10 y	5 (16.7)	15 (53.6)
≥10-<20 y	19 (63.3)	11 (39.3)
≥20 y	6 (20.0)	2 (7.1)
Previous pituitary surgery, n (%)	26 (86.7)	24 (85.7)
Time since surgery, months, median (range)	154.3 (19.2-425.9)	96.8 (19.1-327.1)
IGF-I group at screening, × ULN, n (%)		
<0.86	14 (46.7)	13 (46.4)
≥0.86	16 (53.3)	15 (53.6)
Baseline IGF-I,*^a^* ×ULN, mean (SD)	0.83 (0.14)	0.82 (0.16)
Screening GH,*^b^* ng/mL, mean (SD)	0.92 (1.02)	0.89 (0.83)
Prior injected SRL, n (%)		
Octreotide long-acting release	18 (60.0)	16 (57.1)
Lanreotide depot	12 (40.0)	12 (42.9)
Prior injected octreotide dose, n (%)		
Low (10 mg/mo)	1 (3.3)	2 (7.1)
Mid (20 mg/mo)	7 (23.3)	11 (39.3)
High (30 or 40 mg/mo)	10 (33.3)	3 (10.7)
Prior injected lanreotide dose, n (%)		
Low (60 mg/mo or 120 mg/8 wk)	2 (6.7)	1 (3.6)
Mid (90 mg/mo or 120 mg/6 wk)	4 (13.3)	5 (17.9)
High (120 mg/mo)	6 (20.0)	6 (21.4)

Abbreviations: SRL, somatostatin receptor ligand; ULN, upper limit of normal.

^*a*^Mean of screening and day 1 IGF-I values.

^*b*^Mean from 5 samples collected at least 30 minutes apart over a 3-hour period; screening served as baseline value.

### Biochemical Control

**Figure 3.**
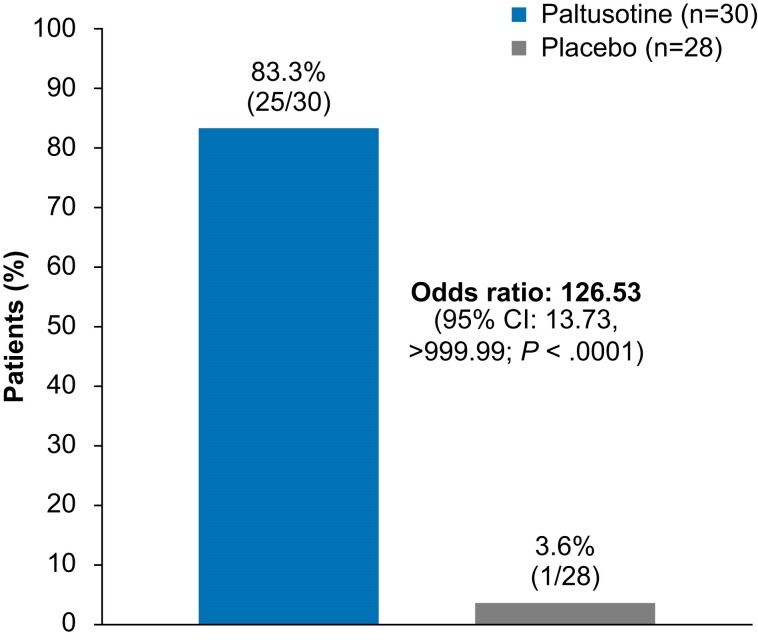
Primary endpoint: proportion of patients with IGF-I ≤1.0× ULN at the end of the randomized controlled phase. The primary endpoint was based on average of IGF-I values at weeks 34 and 36. Odds ratio is from an exact logistic regression model with categorical covariates of prior treatment (octreotide, lanreotide) and screening IGF-I level (<0.86× ULN, ≥0.86× ULN).

**Figure 4.**
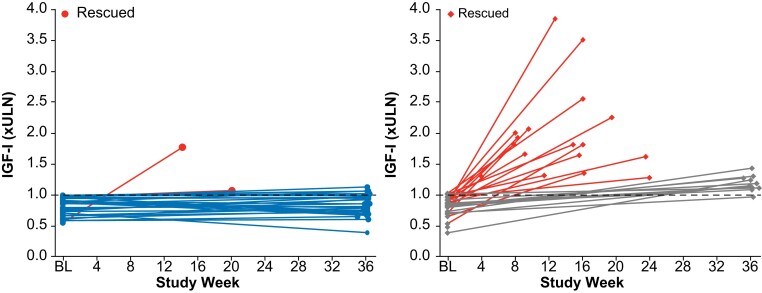
IGF-I levels at baseline and end of treatment (week 36 or last assessment before rescue) for individual patients. Red lines represent patients who received rescue medication; blue (paltusotine) and gray (placebo) lines represent patients who completed the core phase without rescue.

Abbreviation: ULN, upper limit of normal.

In the primary efficacy analysis, 83.3% (25/30) of patients in the paltusotine group met the primary endpoint, compared with 3.6% (1/28) of patients in the placebo group (odds ratio [OR], 126.53; 95% CI, 13.73->999.99; *P* < .0001; Figs. 3 and 4). Sensitivity analyses supported the primary analysis. Among week 34 completers, the proportion of patients meeting the primary endpoint was 89.3% (25 of 28) in the paltusotine group and 9.1% (1 of 11) in the placebo group (OR, 65.39; 95% CI, 11.33->999.99; *P* < .0001). In the per-protocol population, the primary endpoint was met by 83.3% (25 of 30) and 3.7% (1 of 27) of patients, respectively (OR, 118.43; 95% CI, 13.01->999.99; *P* < .0001). In addition, rates of IGF-I control were similar in patients previously treated with injected octreotide (paltusotine 83.3% vs placebo 0%) or lanreotide (83.3% vs 8.3%).

**Figure 5.**
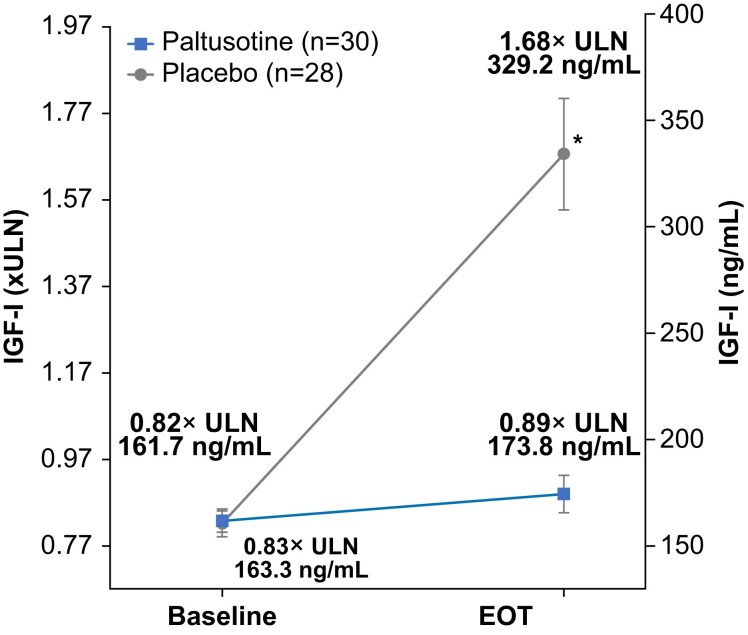
Mean IGF-I level at baseline and end of treatment. *P < .0001 for paltusotine vs placebo. Error bars indicate standard error.

Abbreviation: ULN, upper limit of normal.

Median time to IGF-I >1.0× ULN was 84 days (interquartile range, 58-85 days) in the placebo group; median time in the paltusotine group was not reached because few patients experienced IGF >1.0× ULN. At the end of study treatment, mean (±SE) change from baseline IGF-I level (0.04 ± 0.09× ULN with paltusotine vs 0.83 ± 0.1× ULN with placebo) differed significantly between groups (treatment difference, −0.79; 95% CI, −1.06 to −0.53; *P* < .0001), meeting the secondary endpoint (Fig. 5).

**Figure 6.**
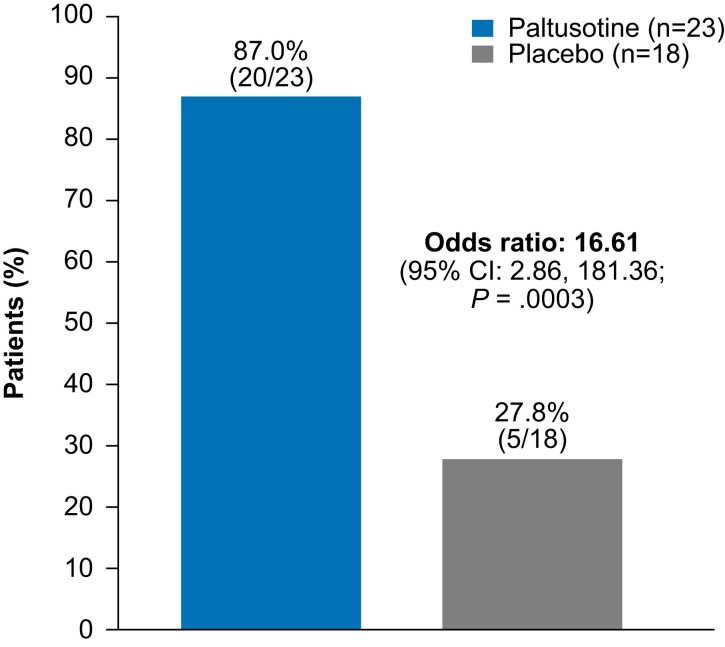
Proportion of patients with mean 5-sample GH <1.0 ng/mL at screening who maintained mean 5-sample GH <1.0 ng/mL at week 34 (secondary endpoint). Odds ratio is from an exact logistic regression model with categorical covariates of prior treatment (octreotide, lanreotide) and screening IGF-I level (<0.86× ULN, ≥0.86× ULN).

Similar to the findings for IGF-I, least-squares mean (±SE) change in 5-sample GH at the end of randomized treatment was −0.24 ± 0.30 ng/mL in the paltusotine group and 1.72 ± 0.31 ng/mL in the placebo group (treatment difference, −1.96; 95% CI, −2.82 to −1.10; *P* < .0001). The proportion of patients who maintained 5-sample mean GH <1.0 ng/mL at week 34 was evaluated in the 41 patients with 5-sample GH <1.0 ng/mL at screening (secondary endpoint); a significantly greater proportion (87.0% vs 27.8%; *P* = .0003) remained below this threshold with paltusotine vs placebo (Fig. 6).

The rate of rescue medication use was significantly lower for paltusotine (2 patients [6.7%]) vs placebo (17 patients [60.7%]; OR, 0.05; 95% CI, 0.005-0.27; *P* < .0001). In 1 patient receiving paltusotine, IGF-I was 1.77× ULN at the time of rescue and, for unknown reasons, remained elevated 5 months after prior injected SRL medication was restarted. The other paltusotine-treated patient did not meet the protocol-specified criteria for rescue (IGF- I of 1.08× ULN) but was experiencing AEs of arthralgia, dyspnea, and fatigue, which were rated as mild and deemed unrelated to study drug by the investigator; rescue medication was nevertheless administered at the investigator's discretion.

### Symptom Control

**Figure 7.**
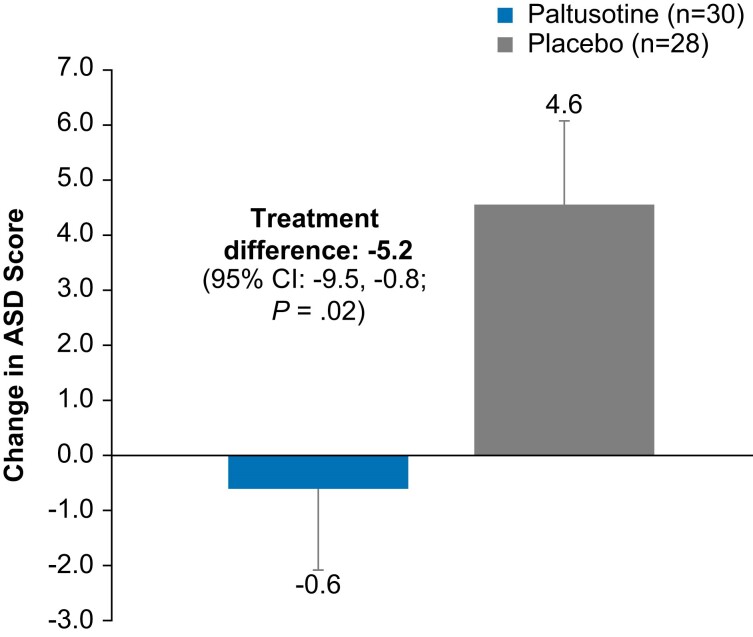
Least-squares mean change in ASD total score from baseline at end of treatment (week 36 or last assessment before rescue) (secondary endpoint). Higher scores indicate worsening. Change from baseline values (reported as least-squares means) and treatment difference are from analysis of covariance. Error bars indicate standard error. Abbreviation: ASD, Acromegaly Symptom Diary.

**Figure 8.**
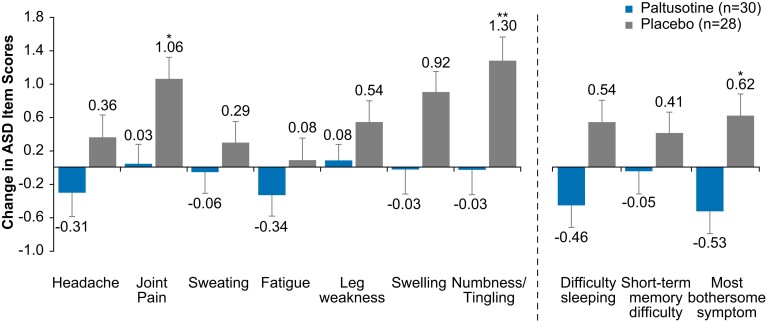
Least-squares mean change in ASD item scores from baseline at end of treatment (exploratory endpoint). Higher scores indicate worsening. Dashed line separates core items (left) from additional items and most bothersome symptom (right). Change from baseline values (reported as least-squares means) and *P* values are from analyses of covariance. Error bars indicate standard error. *P < .05 for paltusotine vs placebo; **P < .01 for paltusotine vs placebo. Abbreviation: ASD, Acromegaly Symptom Diary.

The mean completion rate of daily ASD questionnaires was 79.1%. At the end of randomized treatment, the ASD total score was significantly increased from baseline (indicating worsening) in the placebo group compared with the paltusotine group (least-squares mean [±SE] change, 4.6 [1.6] vs −0.6 [1.5]; *P* = .02), meeting the secondary endpoint (Fig. 7). For individual ASD symptoms, mean scores were generally unchanged with paltusotine and indicative of worsening with placebo; the between-group difference was statistically significant for joint pain, numbness/tingling, and the most bothersome symptom (Fig. 8).

### Safety

Mean rates of medication adherence were 97.9% and 99.6% in the paltusotine and placebo groups, respectively. Overall, 24 (80.0%) patients in the paltusotine group and 28 (100%) in the placebo group experienced ≥1 AE (Table 2). The most commonly reported AEs in paltusotine-treated patients were arthralgia, headache, diarrhea, and abdominal pain, all of which were mild to moderate in intensity. The gastrointestinal AEs were resolved without discontinuing paltusotine, consistent with the typical course of SRL-related gastrointestinal side effects (10). The incidence of AEs classified by the investigator as related to acromegaly was lower for paltusotine compared with placebo (30.0% and 85.7%, respectively).

**Table 2.** Adverse events occurring in ≥5% of patients in either randomized treatment group during the core phase

Adverse events, n (%)*^a^*	Paltusotine (n = 30)		Placebo (n = 28)	
**Summary of adverse events**				
Any	24 (80.0)		28 (100)	
Severe***^b^***	0		3 (10.7)	
Serious***^c^***	0		1 (3.6)	
Led to discontinuation of treatment*^d^*	2 (6.7)		17 (60.7)	
**Common signs and symptoms of acromegaly**	**Overall**	**Attributed to acromegaly** *^e^*	**Overall**	**Attributed to acromegaly** *^e^*
Arthralgia	9 (30.0)	5 (16.7)	16 (57.1)	14 (50.0)
Headache	6 (20.0)	5 (16.7)	10 (35.7)	8 (28.6)
Peripheral swelling	2 (6.7)	1 (3.3)	10 (35.7)	9 (32.1)
Fatigue	2 (6.7)	1 (3.3)	5 (17.9)	5 (17.9)
Asthenia	1 (3.3)	0	4 (14.3)	2 (7.1)
Muscle weakness	1 (3.3)	0	3 (10.7)	2 (7.1)
Paresthesia	0	0	7 (25.0)	5 (17.9)
Hyperhidrosis	0	0	4 (14.3)	4 (14.3)
Pain in extremity	0	0	3 (10.7)	2 (7.1)
Back pain	1 (3.3)	0	2 (7.1)	1 (3.6)
**Gastrointestinal adverse events**				
Diarrhea	7 (23.3)		4 (14.3)	
Abdominal pain	5 (16.7)		3 (10.7)	
Nausea	4 (13.3)		2 (7.1)	
Abdominal distension	2 (6.7)		1 (3.6)	
Constipation	2 (6.7)		4 (14.3)	
Decreased appetite	2 (6.7)		0	
Flatulence	2 (6.7)		1 (3.6)	
Gastroenteritis	2 (6.7)		0	
Upper abdominal pain	0		2 (7.1)	
**Other adverse events**				
Iron deficiency anemia	2 (6.7)		0	
Nasopharyngitis	2 (6.7)		1 (3.6)	
Palpitations	2 (6.7)		0	
Urinary tract infection	2 (6.7)		2 (7.1)	
Dizziness	1 (3.3)		2 (7.1)	
Dyspnea	1 (3.3)		2 (7.1)	
Hyperglycemia	1 (3.3)		2 (7.1)	
COVID-19	0		6 (21.4)	

Adverse events were coded using MedDRA version 24.0.

^*a*^Defined as adverse events that emerged or worsened during study treatment, including events with onset after rescue medication was administered.

^*b*^Three patients in the placebo group experienced a severe adverse event: arthralgia in 1 patient; leukopenia in 1 patient; and peripheral swelling, fatigue, and pain in extremity in 1 patient.

^*c*^Serious adverse event of acute cholecystitis in the placebo group, which was judged by the study investigator as not treatment-related.

^*d*^Patients who received rescue medication were considered to have discontinued study treatment because of adverse events (worsening of acromegaly symptoms).

^*e*^Classified by the investigator as related to acromegaly.

For patients with a visible tumor at baseline, median (range) tumor volume was 211.0 mm^3^ (19.0-1249.0) and 296.5 mm^3^ (9.0-9326.0) in the paltusotine (n = 21) and placebo (n = 20) groups, and median (range) percent change at the end of the core phase was 0.0% (−23.2 to 10.8) (n = 18) and 0.0% (−15.4 to 0.0) (n = 17), respectively. No patient had a newly visible tumor at the end of the core phase, and there were no tumor volume increases or decreases of >25%, nor any change deemed clinically significant.

There were no safety findings of concern with respect to changes in heart rate, blood pressure, hemoglobin A1c, glucose, QTc interval, or gallbladder ultrasound in paltusotine-treated patients.

## Discussion

This randomized, placebo-controlled trial met the primary endpoint and all secondary endpoints, demonstrating that once-daily oral paltusotine maintained biochemical and symptom control in patients with acromegaly whose disease was previously controlled with injected SRLs. This is the first demonstration that a highly specific SST2 agonist is effective in the treatment of acromegaly.

In addition to SST2 selectivity, paltusotine was designed to be a once-daily oral alternative to parenterally administered peptide SRLs, with a low risk of toxicity or substantial drug–drug interactions (17). The high rates of IGF-I and GH control being maintained with paltusotine in the present study may relate to efficient absorption of paltusotine from the gastrointestinal tract (bioavailability of 70% for oral solution (23)), as well as its pharmacologic potency for activating the SST2 receptor (EC_50_ = 0.25 nM) (17).

Prior studies have examined biochemical control when switching from 1 SRL therapy to another. In a prospective, open-label study of patients with acromegaly who switched from short-acting subcutaneous octreotide to intramuscular depot octreotide injections, IGF-I was in the normal range in 63.1% of patients before the switch and 65.8% of patients afterward (24). An oral formulation of octreotide, which uses a permeabilizing agent to facilitate intestinal absorption, has been approved for the treatment of acromegaly (25, 26). Oral octreotide is taken twice daily, at least 1 hour before or at least 2 hours after meals. The bioavailability of oral octreotide is estimated to be 0.7% (25, 27). In a 36-week, randomized placebo-controlled study in patients with acromegaly controlled on injected SRL therapy, IGF-I was maintained at or below the upper limit of normal in 58.2% of patients treated with oral octreotide and 19.4% who received placebo (28). A novel formulation of subcutaneously injected depot octreotide (CAM2029) is in late-stage development, with a phase 3 trial finding that 72.2% of patients receiving CAM2029 and 37.5% receiving placebo maintained IGF-I control after 24 weeks of treatment (29).

In the current PATHFNDR-1 study, IGF-I rose above the reference range in all but 1 patient in the placebo group as previous depot medications washed out, which is consistent with the reported rate of IGF-I elevation observed in a 6- to 9-month time frame in a study of patients who discontinued injected long-acting octreotide (30). Only 1 patient receiving paltusotine met protocol criteria for IGF-I elevation (>1.3× ULN) and acromegaly symptom worsening, warranting rescue SRL therapy. Of the 4 additional patients in the paltusotine group who ended treatment with IGF-I values above the ULN, all demonstrated minor variations relative to baseline (IGF-I ≥0.95× ULN on injected SRLs and ≤1.11× ULN at end of paltusotine treatment).

IGF-I levels provide the most useful objective measure of disease activity, but acromegaly is also characterized by its phenotype and associated symptom burden (1). To assess disease-related symptoms, a novel patient-reported outcome tool, the ASD, was developed in accordance with US Food and Drug Administration guidance (22). Data collected in phase 2 clinical studies of paltusotine showed that the ASD had suitable psychometric properties of internal consistency, test-retest reliability, baseline construct validity, and responsiveness to change (22). Preliminary analyses based on these data found that a difference of 4 to 6 points corresponded to a clinically meaningful change in symptoms (22). Thus, the mean treatment difference of 5.2 points in the current study suggests clinically meaningful, as well as statistically significant, deterioration in acromegaly symptoms in the placebo group relative to continued treatment with paltusotine.

The observed safety profile of paltusotine was consistent with the known effects of octreotide and lanreotide. No new or unexpected safety findings of concern were observed, including no clinically significant pituitary tumor size increases during this 36-week study. Paltusotine treatment was associated with lower acromegaly symptom burden, as assessed using the patient-reported ASD, and with lower rates of adverse events attributed by investigators as related to acromegaly.

Strengths of paltusotine include once-daily oral administration, precluding the need for injections and their associated complications and burden. The faster pharmacokinetic washout of an oral medication may be helpful in certain circumstances (eg, managing gastrointestinal side effects at therapy initiation, evaluating for remission following pituitary radiotherapy, or in the event of pregnancy while on medical therapy). A daily oral medication also permits rapid up-titration (paltusotine reaches steady state in 3-5 days (23)), in contrast to monthly SRL injections, for which dose change assessments are made after 3 administrations to allow steady state to be reached. Additionally, daily dosing may be helpful for patients experiencing variable IGF-I and symptom control with monthly SRL injections (12, 15).

Limitations of this trial include the study population, which was restricted to patients with biochemical control on injected SRL monotherapy and is not representative of patients with uncontrolled acromegaly or those who require a regimen of multiple medications to attain adequate disease control. Because this placebo-controlled study was designed to rescue patients early in the course of evolving inefficacy, the increases in IGF-I level and ASD score reported for the placebo group (at the time of rescue) underestimate the deterioration in disease control that would be observed if patients remained untreated. Although the ASD was designed to meet US Food and Drug Administration specifications, it is a relatively new patient-reported outcome measure and has not yet been used in studies of other acromegaly medications. Another potential limitation is that the 36-week duration of the randomized controlled phase may not be sufficient to detect changes in pituitary tumor size. Pituitary tumor stability has been observed in a cohort of patients treated with paltusotine for up to 2 years in a phase 2 extension study (31).

In conclusion, this study demonstrated maintenance of biochemical and symptom control in patients with acromegaly who switched from injected SRLs to paltusotine. Paltusotine was well tolerated and is a promising once-daily oral treatment option for patients with acromegaly. This novel compound demonstrates that oral nonpeptide molecules can modulate G protein–coupled receptors, illustrating the potential for a range of nonpeptide alternatives to injected therapies.

## Data Availability

Some or all datasets generated during and/or analyzed during the current study are not publicly available but are available from the corresponding author on reasonable request.

## Abbreviations

AE: adverse event
ASD: Acromegaly Symptom Diary
OR: odds ratio
PATHFNDR-1: Paltusotine Acromegaly THerapy Featuring a Non-invasive Daily Regimen
SRL: somatostatin receptor ligand
SST2: somatostatin receptor 2
ULN: upper limit of normal

## References

[1] Colao A, Grasso LFS, Giustina A, et al Acromegaly. Nat Rev Dis Primers. 2019;5(1):20. 30899019 10.1038/s41572-019-0071-6

[2] Melmed S . Pituitary-tumor endocrinopathies. N Engl J Med. 2020;382(10):937‐950. 32130815 10.1056/NEJMra1810772

[3] Katznelson L, Laws ER Jr, Melmed S, et al Acromegaly: an Endocrine Society clinical practice guideline. J Clin Endocrinol Metab. 2014;99(11):3933‐3951. 25356808 10.1210/jc.2014-2700

[4] Giustina A, Barkhoudarian G, Beckers A, et al Multidisciplinary management of acromegaly: a consensus. Rev Endocr Metab Disord. 2020;21(4):667‐678. 32914330 10.1007/s11154-020-09588-z PMC7942783

[5] Fleseriu M, Biller BMK, Freda PU, et al A Pituitary Society update to acromegaly management guidelines. Pituitary. 2021;24(1):1‐13. 33079318 10.1007/s11102-020-01091-7 PMC7864830

[6] Starnoni D, Daniel RT, Marino L, Pitteloud N, Levivier M, Messerer M. Surgical treatment of acromegaly according to the 2010 remission criteria: systematic review and meta-analysis. Acta Neurochir (Wien). 2016;158(11):2109‐2121. 27586125 10.1007/s00701-016-2903-4

[7] Theodoropoulou M, Stalla GK. Somatostatin receptors: from signaling to clinical practice. Front Neuroendocrinol. 2013;34(3):228‐252. 23872332 10.1016/j.yfrne.2013.07.005

[8] Gadelha MR, Wildemberg LE, Bronstein MD, Gatto F, Ferone D. Somatostatin receptor ligands in the treatment of acromegaly. Pituitary. 2017;20(1):100‐108. 28176162 10.1007/s11102-017-0791-0

[9] Gomes-Porras M, Cárdenas-Salas J, Álvarez-Escolá C. Somatostatin analogs in clinical practice: a review. Int J Mol Sci. 2020;21(5):1682. 32121432 10.3390/ijms21051682 PMC7084228

[10] Gadelha MR, Wildemberg LE, Kasuki L. The future of somatostatin receptor ligands in acromegaly. J Clin Endocrinol Metab. 2022;107(2):297‐308. 34618894 10.1210/clinem/dgab726 PMC8764337

[11] Geer EB, Sisco J, Adelman DT, et al Patient reported outcome data from acromegaly patients treated with injectable somatostatin receptor ligands (SRLs) in routine clinical practice. BMC Endocr Disord. 2020;20(1):117. 32736547 10.1186/s12902-020-00595-4 PMC7393879

[12] Strasburger CJ, Karavitaki N, Störmann S, et al Patient-reported outcomes of parenteral somatostatin analogue injections in 195 patients with acromegaly. Eur J Endocrinol. 2016;174(3):355‐362. 26744896 10.1530/EJE-15-1042 PMC4722610

[13] Boyd AE, DeFord LL, Mares JE, et al Improving the success rate of gluteal intramuscular injection. Pancreas. 2013;42(5):878‐882. 23508015 10.1097/MPA.0b013e318279d552

[14] Debono M, Hon LQ, Bax N, Blakeborough A, Newell-Price J. Gluteal nodules in patients with metastatic midgut carcinoid disease treated with depot somatostatin analogs. J Clin Endocrinol Metab. 2008;93(5):1860‐1864. 18303072 10.1210/jc.2008-0019

[15] Maione L, Albrici C, Grunenwald S, et al IGF-I variability over repeated measures in patients with acromegaly under long-acting somatostatin receptor ligands. J Clin Endocrinol Metab. 2022;107(9):e3644‐e3653. 35772775 10.1210/clinem/dgac385

[16] Gadelha MR, Gadelha AC, Kasuki L. New treatments for acromegaly in development. J Clin Endocrinol Metab. 2023;108:e148‐e159. 37757837 10.1210/clinem/dgad568 PMC10940255

[17] Zhao J, Wang S, Markison S, et al Discovery of paltusotine (CRN00808), a potent, selective, and orally bioavailable non-peptide SST2 agonist. ACS Med Chem Lett. 2023;14(1):66‐74. 36655128 10.1021/acsmedchemlett.2c00431 PMC9841592

[18] Gadelha MR, Gordon MB, Doknic M, et al ACROBAT Edge: safety and efficacy of switching injected SRLs to oral paltusotine in patients with acromegaly. J Clin Endocrinol Metab. 2023;108(5):e148‐e159. 36353760 10.1210/clinem/dgac643 PMC10099171

[19] Luo R, Burke G, Mui C, et al Pharmacokinetics and safety of an improved oral formulation of paltusontine, a selective, non-peptide somatostatin receptor 2 (SST2) agonist for the treatment of acromegaly [abstract]. J Endocrine Soc. 2021;5(Suppl 1):A524.

[20] Bidlingmaier M, Friedrich N, Emeny RT, et al Reference intervals for insulin-like growth factor-1 (IGF-I) from birth to senescence: results from a multicenter study using a new automated chemiluminescence IGF-I immunoassay conforming to recent international recommendations. J Clin Endocrinol Metab. 2014;99(5):1712‐1721. 24606072 10.1210/jc.2013-3059

[21] Manolopoulou J, Alami Y, Petersenn S, et al Automated 22-kD growth hormone–specific assay without interference from Pegvisomant. Clin Chem. 2012;58(10):1446‐1456. 22908135 10.1373/clinchem.2012.188128

[22] Martin S, Bender RH, Krasner A, Marmon T, Monahan M, Nelson L. Development and evaluation of the Acromegaly Symptom Diary. J Patient Rep Outcomes. 2023;7(1):15. 36792844 10.1186/s41687-023-00541-7 PMC9931976

[23] Madan A, Markison S, Betz SF, et al Paltusotine, a novel oral once-daily nonpeptide SST2 receptor agonist, suppresses GH and IGF-1 in healthy volunteers. Pituitary. 2022;25(2):328‐339. 35000098 10.1007/s11102-021-01201-z PMC8894159

[24] Lancranjan I, Atkinson AB. Results of a European multicentre study with Sandostatin LAR in acromegalic patients, Sandostatin LAR Group. Pituitary. 1999;1(2):105‐114. 11081188 10.1023/a:1009980404404

[25] Brayden DJ, Maher S. Transient Permeation Enhancer^®^ (TPE^®^) technology for oral delivery of octreotide: a technological evaluation. Expert Opin Drug Deliv. 2021;18(10):1501‐1512. 34128734 10.1080/17425247.2021.1942838

[26] Melmed S, Popovic V, Bidlingmaier M, et al Safety and efficacy of oral octreotide in acromegaly: results of a multicenter phase III trial. J Clin Endocrinol Metab. 2015;100(4):1699‐1708. 25664604 10.1210/jc.2014-4113

[27] Tuvia S, Atsmon J, Teichman SL, et al Oral octreotide absorption in human subjects: comparable pharmacokinetics to parenteral octreotide and effective growth hormone suppression. J Clin Endocrinol Metab. 2012;97(7):2362‐2369. 22539587 10.1210/jc.2012-1179

[28] Samson SL, Nachtigall LB, Fleseriu M, et al Maintenance of acromegaly control in patients switching from injectable somatostatin receptor ligands to oral octreotide. J Clin Endocrinol Metab. 2020;105(10):e3785‐e3797. 32882036 10.1210/clinem/dgaa526 PMC7470473

[29] Ferone D. A randomized phase 3 trial to assess efficacy and safety of a novel formulation of octreotide subcutaneous depot in patients with acromegaly. Presented at the 21st International Congress of Endocrinology; 2024, Dubai, UAE.

[30] Casagrande A, Bronstein MD, Jallad RS, et al Long-term remission of acromegaly after octreotide withdrawal is an uncommon and frequently unsustainable event. Neuroendocrinology. 2017;104(3):273‐279. 27161443 10.1159/000446542

[31] Gadelha MR, Randeva H, Gordon MB, et al Oral, once-daily, paltusotine (non-peptide selective somatostatin receptor subtype 2 agonist) therapy in patients with acromegaly is associated with long-term biochemical and symptom control and is preferred over injectable somatostatin-receptor ligands. Presented at the Endocrine Society's Annual Meeting (ENDO); 2023, Chicago, IL.

